# Sharing more than friendship – transmission of NDM-5 ST167 and CTX-M-9 ST69 *Escherichia coli* between dogs and humans in a family, Finland, 2015

**DOI:** 10.2807/1560-7917.ES.2018.23.27.1700497

**Published:** 2018-07-05

**Authors:** Thomas Grönthal, Monica Österblad, Marjut Eklund, Jari Jalava, Suvi Nykäsenoja, Katariina Pekkanen, Merja Rantala

**Affiliations:** 1 Faculty of Veterinary Medicine, University of Helsinki, Helsinki, Finland; 2 Sydspetsens miljöhälsa, Hangö, Finland; 3 National Institute for Health and Welfare, Turku, Finland; 4 Food Safety Authority Evira, Helsinki, Finland

**Keywords:** carbapenemase, zoonoses, antimicrobial resistance, antimicrobial use

## Abstract

Carbapenemase-producing *Enterobacteriaceae* (CPE) have rarely been reported in dogs, and never in animals in Finland. However, in April 2015, two meropenem-resistant *Escherichia coli* were identified from two dogs in one family. Both dogs suffered from chronic *otitis externa*. **Methods:** Epidemiological and molecular investigations (pulsed-field gel electrophoresis (PFGE), multilocus sequence typing) were conducted to investigate the source of infection and transmission routes. **Results:** In both dogs and one family member New Delhi metallo-beta-lactamase (NDM-5)-producing multidrug-resistant ST167 *E. coli* was found. Whole genome sequencing confirmed that the isolates were identical or only had one or two allelic differences. Additionally, the dogs and humans of the family carried an identical extended-spectrum beta-lactamase (ESBL) CTX-M-group 9 *E. coli* ST69 strain, indicating interspecies transmission. While the original source remains unclear, human-to-canine transmission is possible. No carbapenems had been administered to the dogs, but exposure to numerous other antimicrobials likely sustained the bacteria and supported its propagation in the canine host. **Conclusion:** To our knowledge, canine clinical NDM-5 *E. coli* in Europe, and confirmed CPE transmission between dogs and humans have not been previously reported. The screening of veterinary *Enterobacteriaceae* isolates for carbapenem resistance is highly recommended.

## Introduction

The rapid global emergence of resistance to carbapenem and other extended-spectrum beta-lactams in *Enterobacteriaceae*, *Acinetobacter* and *Pseudomonas* species in the past decade is a major public health concern [1,2]. With such types of resistance, these species have the potential to cause severe infection, especially in hospitalised individuals [3]. Carbapenem resistance is mediated by different types of carbapenemases, of which *Klebsiella pneumoniae* carbapenemase (KPC-type) and New Delhi metallo-beta-lactamase (NDM-type) carbapenemase are the most common in bacteria from human clinical specimens [2]. In addition to resistance to nearly all beta-lactams, isolates carrying carbapenemases are often multi- to extensively drug-resistant (MDR or XDR), leaving very few or no treatment options [4]. Carbapenemase-producing MDR bacteria were first described almost exclusively in humans, but since 2011 have also been detected in livestock, companion animals, wildlife, and different environmental compartments [5,6], indicating their transfer to new hosts and reservoirs. Carbapenem use in humans has been implicated as a cause of emerging carbapenem resistance [7]. Due to the MDR/XDR character of carbapenemase-producing bacteria, carbapenem resistance may also be co-selected for by the use of other antimicrobial classes [8]. Carbapenems are not authorised for veterinary use in Finland (Government decree 1054/2014) [9].

In Finland, carbapenemase-producing bacteria have been found in humans, although cases are still rare, with 20–34 new cases annually in 2013–16. Over half of these were probably of foreign origin [10]. However, such bacteria have never been identified in animals in the country. In the spring of 2015, meropenem-resistant *Escherichia coli* was diagnosed from two canine ear specimens in the Clinical Microbiology Laboratory (CML) of the Faculty of Veterinary Medicine, University of Helsinki. The exceptional findings initiated epidemiological and molecular investigations together with the Finnish Food Safety Authority (Evira) and the National Institute of Health and Welfare (THL). Here, we present the investigations and their results.

## Methods

### Epidemiological investigation

In spring 2015, two dogs, belonging to the same family, were found to have meropenem-resistant MDR *E. coli* in clinical specimens from their ears. Written consent for an epidemiological investigation was obtained from the owners. The investigation covered a timeline of up to two years before the findings. The medical records of the dogs were obtained from the treating veterinarian. The owners were asked to fill in an electronic questionnaire that covered their medical history including hospitalisations, human and animal contacts, travel history and the living conditions of the dogs. The family members were also instructed to contact their local healthcare centre to consider screening for carbapenem and extended-spectrum cephalosporin-resistant bacteria. The presence of carbapenemase and other extended-spectrum beta-lactamase-producing bacteria in the canine patients was followed for a year by taking specimens from the dogs’ ears (4 occasions) and rectum (3 occasions). In addition, a retrospective search of the records of the CML and the Reference Laboratory of THL was performed for the presence of respective *E. coli* sequence types (ST) with similar antibiograms to those in this study.

### Microbiological investigation

#### Laboratory methods for canine specimens

Clinical ear specimens were cultured on 5% sheep blood agar (tryptic soy agar with Sheep Blood, Oxoid, United Kingdom (UK)), a chromogenic agar (UriSelect 4, Bio-Rad, France) and Dixon agar plates (Tammertutkan Maljat, Finland). Bacteria were identified using biochemical methods [11], while disk diffusion susceptibility testing, including quality control and interpretation, was performed according to Clinical and Laboratory Standards Institute (CLSI) guidelines with *E. coli* ATCC 25922 as a quality control [12,13]. If no veterinary-specific susceptibility breakpoints were available, human CLSI breakpoints were used [14]. The susceptibility testing panel for *Enterobacteriaceae* included amikacin, amoxicillin-clavulanic acid, ampicillin, cefpodoxime, chloramphenicol, doxycycline, enrofloxacin, gentamicin, meropenem and trimethoprim-sulfamethoxazole (Oxoid, UK). Follow-up screening specimens from the ears and rectum were cultured on a selective agar (Brilliance ESBL, Oxoid, UK) and a blood agar plate with a 10 µg meropenem disk and incubated at + 35 ± 1 °C for up to 48 hours. *Enterobacteriaceae* isolates growing on the selective plate or showing reduced susceptibility to cefpodoxime (≤ 22 mm) or meropenem (≤ 22 mm) were identified and preserved for further research. Preliminary screening of carbapenemase activity was carried out using the modified Hodge test [14]. The type of beta-lactamase produced was confirmed by using commercial disk sets (carbapenemases: KPC +  MBL Confirm ID Kit, Rosco Diagnostica, Denmark; ESBL/AmpC-type beta-lactamases: AmpC and ESBL Detection Set, Mast Group Ltd, UK). Carbapenemase activity was confirmed with ultraviolet-spectrometric detection of imipenem hydrolysis [15].

#### Bacterial strains from humans

Three extended-spectrum beta-lactamase (ESBL)/carbapenemase-producing *E. coli* isolates isolated from rectal swabs of two human family members were received from the Helsinki-Uusimaa Hospital District Laboratory, HUSLAB. The rectal swabs were obtained and the specimens were cultured according to guidelines that have since been published [16]. In addition, susceptibility results were available from a fourth human ESBL *E. coli* isolated from a follow-up screening specimen. Sequence typing of the isolates was conducted as further described. A search from the National Infectious Diseases Register at THL was performed to identify human *E. coli* isolates with similar ST and carbapenemase profiles in Finland in 2014–15. These were included in the core genome multilocus sequence typing (cgMLST) comparison (see below).

### Molecular methods

#### Pulsed-field gel electrophoresis

Pulsed-field gel electrophoresis (PFGE) was performed as previously described [17]. Clonal similarity was determined using the Dice coefficient and the unweighted pair-group method using arithmetic average clustering (UPGMA), with the optimisation and tolerance both set to 1% (GelCompar software version 6, Applied Maths, Belgium).

#### Multilocus sequence typing and detection of beta-lactamase-encoding genes by PCR

DNA for multilocus sequence typing (MLST) and PCR was extracted by suspending one to three bacterial colonies in 200 µL of PCR-grade water. The suspension was boiled for 10 min and centrifuged at 13,000 rpm for 5 min. The supernatant was used as a DNA template.

For MLST, amplification of *adk*, *fumC*, *gyrB*, *icd*, *mdh*, *purA* and *recA* genes was performed using primers by Lau et al. [18], which were attached to universal forward (UniF: 5’-GTTTTCCCAGTCACGACGTTGTA-3’) and reverse (UniR: 5’-TTGTGAGCGGATAACAATTTC-3’) sequences at the 5’ end of each primer. The universal F and R sequences were used as sequencing primers. The PCR reaction mixture (total volume 20 µL) contained 10 µL of 2x Phusion Flash High Fidelity Master Mix (Thermo Scientific, United States (US)), 0.25 µM of each primer (Oligomer Oy, Finland) and 1 µL of DNA template for all reactions, except for *icd*, for which a primer concentration of 0.5 µM was used. PCR conditions for *adk*, *fumC* and *gyrB* were as follows: initial denaturation at 98 °C (15 s); 30 cycles of denaturation at 98 °C (2 s), annealing at 63 °C (10 s), elongation at 72 °C (15 s), and final elongation at 72 °C (60 s). The PCR parameters for *icd*, *mdh*, *purA* and *recA* multiplication were the same, except for an annealing temperature of 59 °C (10 s).

Multiplex PCRs in a Phire Green Hot Start II PCR Master Mix (MM, total volume 20 µL) (Thermo Scientific, US) were performed to screen ESBL (CTX-M, TEM and SHV) and AmpC (CIT, DHA, ACC and FOX) gene families. All primers used in these and in the sequencing of CTX-M, as well as PCR conditions, are presented in Table 1 [19-22]. The reaction mixture for ESBL multiplex PCR and CTX-M sequencing contained 15.4 µL of MM, 0.6 µL of dimethyl sulfoxide (DMSO), 0.25 µM of each primer (Oligomer Oy, Finland) and 1 µL of template DNA. The AmpC multiplex PCR contained 13.8 µL of MM, 0.6 µL of DMSO and 0.25 µM of each primer, except for FOX primers, for which the concentration was 0.4 µM. Carbapenemase gene families (KPC, IMP, VIM, NDM, OXA, GES, and IMI) were detected with multiplex PCR (Table 1) [15]. The reaction mixture contained 0.08 U/μL of AmpliTaq Gold polymerase (Applied Biosystems, US) in a 1x reaction buffer (AmpliTag Gold buffer 10x Mg free, Applied Biosystems, US), 2.0 mM of MgCl_2_, 0.2 mM of nt and 0.3 pmol/μL of each primer. A mixture of DNA from bacterial strains (characterised by THL) carrying either the ESBL (multiplex ESBL PCR), AmpC (multiplex AmpC PCR) or carbapenemase (multiplex carbapenemase) genes mentioned above were used as positive controls in the respective reactions. PCR-grade water was used as a non-template control in each assay.

**Table 1:** Primers for extended-spectrum beta-lactamase, AmpC and carbapenemase multiplex PCRs and for sequencing of CTX-M group

PCRs	Forward (-F) and reverse (-R) primer sequences and orientation (5’ → 3’)	Target	Size (bp)	PCR conditions^a^	Reference
ESBL-multiplex	CTX-F: ATGTGCAGYACCAGTAARGTKATGGC CTX-R: CDCCGCTGCCGGTYTTATCVCC	CTX-M	513	98°C (30s), 98°C (5s)¸ 65°C (15s), 72°C (20s), 72°C (60s)	[19] [20]
	TEM-F: ATTTYCGTGTCGCCCTTATTCC TEM-R: AAGCGGTTAGCTCCTTCGGTC	TEM	431		[20] This study
	SHV-F: ACCAGCCAGCGTCTGAGC SHV-R: TTGCCAGTGCTCGATCAG	SHV	285		This study [21]
AmpC-multiplex	CIT-F: TGGCCAGAACTGACAGGCAAA CIT-R: TTTCTCCTGAACGTGGCTGGC	CIT	462	98°C (30s), 98°C (5s)¸ 65°C (15s), 72°C (20s), 72°C (20s)	[22]
	DHA-F: AACTTTCACAGGTGTGCTGGGT DHA-R: CCGTACGCATACTGGCTTTGC	DHA	405		
	ACC-F: AACAGCCTCAGCAGCCGGTTA ACC:R: TTCGCCGCAATCATCCCTAGC	ACC	345		
	FOX-F: AACATGGGGTATCAGGGAGATG FOX-R: CAAAGCGCGTAACCGGATTGG	FOX	190		
Carbapenemase-multiplex	KPC-for: CTTGCTGCCGCTGTGCTG KPC-rev: GCAGGTTCCGGTTTTGTCTC	KPC	488	94°C (10 min), 94°C (30s)¸ 59°C (30s), 72°C (60s)	[15]
	Imp-F-g-16: GCAGGTTCCGGTTTTGTCTC Imp-R-g13–4: CCAAACyACTACGTTATCTkGAG	IMP	232		
	Vim-F: GATGGTGTTTGGTCGCATA Vim-R: CGAATGCGCAGCACCAG	VIM	389		
	NDM-for: GGCAGCACACTTCCTATCTC NDM-rev: GTTGATCTCCTGCTTGATCC	NDM	155		
	OXA-48A: TTGGTGGCATCGATTATCGG OXA-48B: GAGCACTTCTTTTGTGATGGC	OXA	743		
	GES-1A: ATGCGCTTCATTCACGCAC GES-1B: CTATTTGTCCGTGCTCAGG	GES	863		
	IMI-A: ATAGCCATCCTTGTTTAGCTC IMI-B: TCTGCGATTACTTTATCCTC	IMI	818		
CTX-M gene grouping^b^	CTX-F: ATGTGCAGYACCAGTAARGTKATGGC CTX-R: CDCCGCTGCCGGTYTTATCVCC	CTX-M	513	98°C (30s), 98°C (5s)¸ 65°C (10s), 72°C (15s), 72°C (60s)	[19] [20]

ESBL: extended-spectrum beta-lactamase.

^a^ Conditions for ESBL-, AmpC multiplex PCRs and for CTX-M-gene PCRs are reported in the following order: initial denaturation, denaturation, annealing, extension (30 cycles), and final extension.

^b^ The same primers were used for PCR and sequencing.

For sequencing of MLST genes and CTX-M, the PCR products were purified with *Exo*I and FastAP (Thermo Scientific, US). Sequencing was performed at Macrogen Inc. (the Netherlands) using an ABI 3730 XL automated sequencer. MLST sequences were analysed using CLC Main Workbench (version 6.9.1, Qiagen, Denmark) and the CLC MLST module (version 1.4.7, Qiagen, Denmark). CTX-M sequencing data were analysed using CLC Main Workbench (version 7.5.1) and compared with the National Center for Biotechnology Information (NCBI) library.

#### Whole genome sequencing

DNA was extracted using the MagAttract HMW DNA Kit (Qiagen, Germany). Whole genome sequencing (WGS) was performed for carbapenem-resistant isolates using an Illumina MiSeq sequencer (Illumina Inc., US) with the Illumina Nextera XT DNA Sample Preparation Kit, the Nextera XT Index Kit with 24 indices for library preparation and MiSeq Reagent Kit V2 (300 cycles) using 150-bp paired-end sequencing. The reads were compiled using Velvet assembler version 1.1.04 included in Ridom SeqSphere + software (Ridom SeqSphere + version 2.4.0; Ridom GmbH, Germany). Beta-lactamase resistance genes were detected using ResFinder 2.1 on the Center for Genomic Epidemiology (CGE) server [23]. Plasmids were detected using PlasmidFinder 1.3 [24]. 

An *ad hoc* core-genome MLST (cgMLST) protocol for *E. coli,* containing 2,634 target alleles, was used on SeqSphere + to compare the strains originating from the family (i.e. the dogs and owners) to strains previously isolated from human patients in Finland, with similar ST and carbapenemase profile, which were identified from the National Infectious Diseases Register at THL. The *ad hoc* cgMLST scheme was created using the cgMLST Target Definer of Ridom SeqSphere software and genome sequences obtained from GenBank. The whole-genome sequence of *Escherichia coli* strain G150 (GenBank accession number: LQHK01000008) was used as a reference strain and three *E. coli* strains, TW14588 (GenBank accession number: NZ_CM000662), 453 (GenBank accession number: NZ_MPGR01000001) and 8368 (GenBank accession number: NZ_CP017444), were used as query genomes. 

## Results

### Case presentations

The patients were two male Finnish Hound dogs, Dog A (five years-old) and Dog B (two-years old), living in the same family. Both dogs had a long history of recurrent *otitis externa*, the underlying factor of which was unidentified, although allergy/atopy was suspected. Dog A had its first *otitis externa* episode in February 2013, when a local veterinarian diagnosed ear inflammation with yeast overgrowth in the dog’s right ear. Dog B began experiencing ear problems in October 2013. Both dogs continued to experience intermittent symptoms of ear infection, despite both topical and systemic antimicrobial (Table 2) and non-antimicrobial therapy, including corticosteroids. 

**Table 2:** Antimicrobial treatments^a^ the dogs had received before the NDM-*Escherichia coli* finding, Finland, 2013–2015

Dog A		Dog B	
Topical	Systemic	Topical	Systemic
Gentamicin Framycetin Fusidic acid Marbofloxacin Miconazole Polymyxin B	Amoxicillin-clavulanic acid Cefalexin Enrofloxacin Itraconazole	Fusidic acid Framycetin Marbofloxacin Miconazole Polymyxin B	Cefalexin Clindamycin Enrofloxacin Fluconazole Itraconazole

NDM: New-Delhi metallo-beta-lactamase producing.

^a^ In addition, the dogs had received local and systemic corticosteroids.

**Figure 1:**
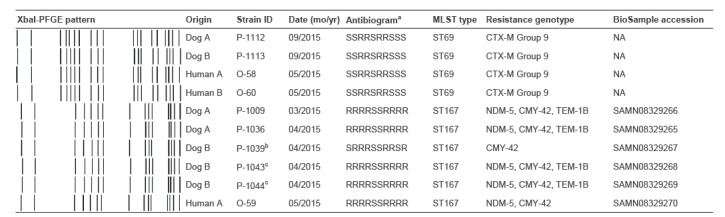
Pulsed-field gel electrophoresis cluster analysis of the ESBL- and NDM- *Escherichia coli* strains isolated from two dogs and their owners, Finland, March 2015–September 2016 (n = 10 isolates)

ESBL: extended-spectrum beta-lactamase; MLST: multilocus sequence typing; NA: not available; NDM: New-Delhi metallo-beta-lactamase producing.

^a^ Antibiogram is represented in the following order: amikacin, amoxicillin-clavulanic acid, ampicillin, cefpodoxime, chloramphenicol, doxycycline, enrofloxacin, gentamicin, meropenem, and trimethoprim-sulfamethoxazole.

^b^ P-1039 is meropenem susceptible MDR-*E. coli*.

^c^ These two strains were otherwise identical, but P-1043 had a rough colony type.

*Otitis media* was diagnosed in the left ear of Dog A in November 2014, while the same condition had been diagnosed in the left ear of Dog B in December 2013. In March 2015, a bacteriological specimen from the left ear of Dog A was sent to the CML. The specimen revealed a meropenem-resistant MDR *E. coli* (for resistance profile, see Figure 1), but carbapenemase production, according to the modified Hodge’s test, appeared to be negative. At that time, the *E. coli* isolate was preserved for further investigation due to the unusual resistance profile. The veterinarian was informed about the unusual phenotype and was advised to handle the dog with contact isolation precautions. Specimens from the ears of both dogs were taken in late April 2015. The specimen from Dog A again revealed meropenem-resistant MDR *E. coli*, along with non-specific growth. This *E. coli* isolate gave a positive result for carbapenemase production in the modified Hodge’s test. This isolate and the earlier meropenem-resistant *E. coli* from Dog A were both metallo-beta-lactamase positive in the double-disk diffusion test. Dog B’s specimen initially revealed only MDR *E. coli* that was meropenem sensitive, but after the meropenem-resistant *E. coli* finding from Dog A, Dog B’s specimen was re-cultured onto a chromogenic agar (UriSelect, Bio-Rad, US) on which a meropenem disc was placed. This revealed a meropenem-resistant *E. coli* isolate with the same antibiogram as Dog A’s isolates. Systemic treatment with antimicrobials was stopped and reasonable control of the ear infection in both dogs was achieved by topical chloramphenicol treatment and anti-inflammatory therapy. The specimen histories of the dogs with respective findings are presented in Table 3.

**Table 3:** Timeline and results of ear and screening specimens from dogs A and B, Finland, December 2013–May 2016 (n = 18 specimens)

Dog	Time	Specimen type	Finding	*E. coli* ID-number
A	March 2015	Ear	NDM-*Escherichia coli*	P-1009
	April 2015	Ear	NDM-*E. coli*, CoNS	P-1036
	June 2015	Ear	No growth	NA
	September 2015	Ear	*Moraxella* sp.	NA
		Rectal swab	ESBL-*E. coli*^a^	P-1112
	December 2015	Ear	*Staphylococcus pseudintermedius, Acinetobacter* sp., yeast	NA
		Rectal swab	ESBL-*E. coli*^a^	NA
	May 2016	Ear	Non-specific growth	NA
		Rectal swab	Negative	NA
B	December 2013	Ear	*S. pseudintermedius, Acinetobacter baumannii*	NA
	April 2015	Ear	NDM-*E. coli*; MDR-*E. coli* (MEM-S)	P-1044, (P-1043)^b^, P-1039
	June 2015	Ear	No growth	NA
	September 2015	Ear	*Mucor circinelloides* (fungus)	NA
		Rectal swab	ESBL-*E. coli*	P-1113
	December 2015	Ear	*S. pseudintermedius, Corynebacterium* sp.	NA
		Rectal swab	Negative	NA
	May 2016	Ear	Non-specific growth	NA
		Rectal swab	Negative	NA

CoNS: coagulase-negative staphylococci; MDR: Multidrug resistant; MEM-S: meropenem susceptible; NA: not applicable (no isolate stored); NDM: New-Delhi metallo-beta-lactamase producing,

^a^ Identical resistance profiles.

^b^ P-1043 was identical to P-1044 (antibiogram, pulsed-field gel electrophoresis, multilocus sequence typing), except it had a rough colony type.

### Epidemiological investigation

The dogs were domestic Finnish Hounds from the same family in rural Eastern Finland. The family consisted of only two adults, and the only animals were the dogs in question. Both family members were of Finnish ethnicity. The dogs lived together in a fenced yard with a dog house and were used for hunting hares. Their diet mainly comprised hare and elk offal, in addition to raw commercial dog food (domestic). They also received cooked cow spines (domestic) and, occasionally, leather bones (unknown origin). The dogs were taken to the woods for hunting or training approximately three times per week. The dogs had not travelled outside of Finland and had not been in contact with other animals while at home. However, while hunting, they had contact with wild animals, mainly hares, but probably also with mice and voles. The human family members had visited Estonia, Sweden, Norway and Croatia within the previous 2 years. Their medical history included hospitalisations in Finland, but not abroad. Neither of the dog owners worked in the healthcare sector.

### Bacteriological and molecular investigations

The two human family members were screened on a voluntary basis for the presence of carbapenemase/ESBL-producing Gram-negative rods. One of them carried NDM- as well as ESBL-producing *E. coli*, while the other family member only carried ESBL-producing *E. coli.* Altogether, seven canine and three human *E. coli* isolates were further typed. One isolate (P-1043) from Dog B was identical to isolate P-1044 from the same dog, apart from colony morphology (Figure 1). All meropenem-resistant isolates (n = 5) belonged to ST167, had an identical antibiogram and PFGE profile, showed metallo-beta-lactamase activity, and were positive for NDM, CIT and TEM by multiplex PCR, except for the human isolate O-59, which was TEM-negative. In addition, one canine isolate susceptible to meropenem and amikacin belonged to the same cluster. All ESBL *E. coli* isolates (n = 4) were part of the same PFGE cluster and MLST type (ST69), with a characteristic antibiogram. Sequencing confirmed the presence of a CTX-M group 9 gene in the isolates of the latter cluster (Figure 1).

WGS was performed on five canine ST167 *E. coli* isolates and the human NDM-positive ST167 isolate. Sequences were submitted to the NCBI BioSample database (see Figure 1 for accession numbers). All five meropenem-resistant isolates carried NDM-5. All six isolates had CMY-42 in their WGS profile (Figure 1). 

**Figure 2:**
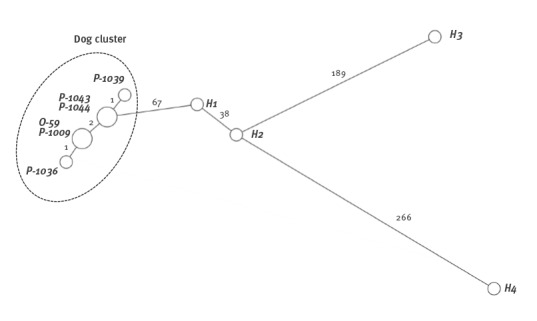
Relatedness of NDM-5 *Escherichia coli* ST167 isolates based on cgMLST analysis, Finland, 2014–2015

*Ad hoc* cgMLST grouped the six isolates very tightly: one strain from Dog A and the owner’s strain were identical, as were two strains from Dog B. Other strains, including one without NDM-5, had only 1 to 2 allelic differences (Figure 2). The search for previous isolates in Finland of the same type and antibiogram as the ones found in the family revealed four human *E. coli* ST167 isolates, all NDM-5 positive, from 2014–15. In the cgMLST analysis these isolates were clearly different from the strains of this study, differing in at least 67 alleles. All the NDM-5-positive *E. coli* strains of this study had *Inc*I1- and *Inc*FII-type plasmids, while the strain without the NDM-5 gene had only *Inc*I1-type plasmids.

cgMLST: core genome multilocus sequence typing; NDM: New-Delhi metallo-beta-lactamase producing.

Minimum spanning tree based on comparing of 2,634 target genes and ignoring missing values in pairwise comparison. The dashed circle defines the strains of this study. Strains H1 to H4 are NDM-5 positive *E. coli* ST167 strains from humans isolated in 2014 and 2015. The numbers above the lines connecting the strains indicate allelic differences. P-1036 and P-1009 are from dog A; P-1044, P-1043, and P-1039 are from Dog B (P-1043 was isolated together with P-1044, was otherwise similar but had a rough colony type). O-59 is from human A. All these were NDM-5 positive except for P-1039.

A retrospective search of the CML database yielded CTX-M-positive *E. coli* ST167 isolates in horses in Finland, but these were vastly different in PFGE compared with the NDM-5 *E. coli* of this study (data not shown). No other previous *E. coli* ST69 of animal origin was observed.

### Follow-up specimens

Carbapenemase-producing *E. coli* was not detected in canine follow-up specimens, but rectal screenings of both dogs in September 2015 yielded CTX-M-positive *E. coli* ST69 (Table 3). Only one follow-up specimen was taken from human A, who originally carried both NDM and CTX-M-positive *E. coli* strains. The follow-up specimen again yielded ESBL *E. coli* with a similar antibiogram to the other ST69 isolates in this study. However, this isolate was not available for typing.

## Discussion

The results from molecular analyses strongly indicate the transmission of ST167 NDM-5 and ST69 CTX-M group 9 *E. coli* between two dogs and humans in the same family. The transfer of ESBL-producing *Enterobacteriaceae* between humans and dogs has previously been described [25], but to our best knowledge, this is the first report on the transmission of carbapenemase-producing *E. coli* between dogs and humans. 

We consider that the transmission of ST167 NDM-5 had most likely occurred from human to dog. There are several reasons for this. Firstly, CPE are more frequent in humans than in animals [6]. Secondly, according to resistance surveillance, carbapenem-resistant bacteria have never been observed in animals in Finland before this report. They have, however, been observed with a gradually increasing frequency in humans, most of whom probably acquired the bacteria abroad [26]. In 2008–15, among 31 Finnish human NDM cases, 11 were NDM-5 *E. coli*. Seven of these had been imported from India, and one each from Croatia, Thailand and Egypt, while one was of unknown origin [26]. The human family members of this study had a history of hospitalisation in Finland, but not abroad. They also had visited Croatia in May 2013, which might be one possible origin for the NDM-5 *E. coli* ST167. The Balkan region has been reported as a potential reservoir of NDM genes, particularly NDM-1 [27]. It is also unlikely that the dogs had acquired NDM *E. coli* from their feed, although it is known that exposure to raw meat increases the risk of ESBL carriage in dogs [28,29]. However, carbapenem-resistant isolates have not been detected in food-producing animals in Finland [30], and even the presence of ESBL/AmpC-type beta-lactamases (CTX-M-1 and CMY-2) is deemed to be at a low level in Finnish livestock (0.8%, 1% and 8.1% in cattle, pigs and broilers, respectively in 2013–14) [31]. No data exist on the prevalence of CPE among wild animals or in the environment in Finland, and these cannot therefore be ruled out as potential sources of infection for the dogs or the owners. However, the general level of resistance in Finnish wild animals was studied in 1999, and was found to be much lower than in the human population [32]. 

The origin of the CTX-M-9 *E. coli* ST69 isolate is unclear. So far, ST69 *E. coli* have not been observed in companion animals in Finland (data not shown), while CTX-M is a common ESBL family among canine ESBL isolates [33]. However, no data are yet available on the genetic variants of canine CTX-M in Finland.

Reports concerning carbapenem-resistant bacteria in companion animals are uncommon [34-42]. In 2013, a US study reported six unrelated NDM *E. coli* isolates collected in 2008–2009 from five canine and one feline patient, of which four were confirmed to be NDM-1 [39]. This is interesting, since the first report of NDM-1 carbapenemase in a *K. pneumoniae* and an *E. coli* isolate from a Swedish human patient of Indian origin was observed in 2008 and published a year later [43]. The first NDM-5-positive *E. coli* of animal origin was reported in an Algerian dog in 2015 [42]. The sequence type of the NDM-5 *E. coli* was ST1284, which is a double locus variant of *E. coli* ST167. In addition, OXA-48 has been observed in *E. coli* in companion animals (dogs and cats) in Algeria (2014–15) [37], the US (2009–13) [35] and France (dogs) (2015) [40], and in *E. coli* and *K. pneumoniae* at a veterinary clinic in Germany (dogs) (2012) [41]. Furthermore, OXA-23 *Acinetobacter* has been reported in dogs in France (2012) [36], VIM-1-producing *K. pneumoniae* in a dog in Spain (2014–15) [38], and IMP-4 *Salmonella* Typhimurium in cats at an animal shelter in Australia (year not specified) [34]. Carbapenem-producing bacteria have also been reported in horses and food-producing animals (NDM in poultry and pigs, OXA-23 in dairy cows), as well as in environmental specimens (2003–13) [6].

It seems that the presence of carbapenemase-producing bacteria in animals is not linked to carbapenem use, since carbapenem products are not authorised for veterinary use [6]. Some off-label use could exist in companion animals according to the cascade principle (Articles 10 and 11 of Directive 2001/82/EC) in the European Union [44], but the extent of such use is unknown. In Finland, however, national legislation prohibits the use of carbapenems in veterinary medicine (Government decree 1054/2014). Our investigation did not reveal any carbapenem use in the dogs of this study, but the selection pressure caused by numerous other antimicrobials was probably enough to co-select carbapenem resistance after the acquisition of carbapenem-resistant MDR *E. coli*. For example, ESBL-producing *Enterobacteriaceae* may be co-selected for by fluoroquinolone therapy [45]. Both dogs had received local as well as systemic fluoroquinolone therapy, which may have contributed to the survival of the MDR NDM-5 *E. coli*.

The NDM-5 *E. coli* isolates in our study belonged to ST167. A database search revealed that only four ST167 NDM-5-positive *E. coli* strains had previously been isolated from humans in Finland. In three cases, the patients had had contact abroad, i.e. Thailand, India and Egypt. The results of cgMLST analyses revealed that previous NDM-5 ST167 *E. coli* were not related to the cluster of this study. Previously, the dissemination of NDM-5 has been linked to *Inc*X3 plasmids [46], while the *Inc*I1 and *Inc*FII plasmids that were present in the NDM-5 isolates of this study have been associated with NDM-1 isolates [47]. *Inc*FII was also identified from all previously isolated NDM-5 ST167 *E. coli* strains that were investigated, although these were not related to the strains from the cases presented here. It is possible that all NDM-5-positive dog-cluster *E. coli* strains carried the NDM-5 gene in *Inc*FII-type plasmids, and the one carbapenem-susceptible strain without NDM-5 had lost this plasmid.

As carbapenemase-producing strains and carbapenem resistance genes spread around the world due to travel and the international trade of food and feed, it is likely that such bacteria will be detected in animals in increasing numbers [5], as has been the case with ESBL/AmpC *Enterobacteriaceae*. Close contact with companion animals increases the likelihood of human-to-animal CPE transmission. Once it has entered the animal population, a CPE can be efficiently co-selected by other antimicrobial classes due to the MDR character of CPE. The widespread use of antimicrobials in companion animals could thus enable them to act as reservoirs for CPE isolates and other resistant bacteria. In our cases, all systemic antimicrobials were discontinued and only topical therapy was used. This may have facilitated the reduction of NDM-5 and ESBL *E. coli* below the level of detection in follow-up specimens. 

An enrichment method for screening would have been more sensitive, but was not used, as it is not recommended by the national guidelines. Furthermore, the use of the European Committee on Antimicrobial Susceptibility Testing (EUCAST) epidemiological cut-off value (ECOFF) for meropenem (< 25 mm) [48] instead of the CLSI meropenem screening breakpoint (≤ 22 mm) might have increased the likelihood of detecting non-wild-type isolates. However, as a clinical laboratory, adjusting breakpoints for this specific case was not considered necessary. Moreover, the meropenem histogram from *E.coli* isolates of the CML revealed that the CLSI breakpoint is capable of satisfactorily differentiating non-wild-type isolates from the wild-type population.

In conclusion, to our knowledge, these are the first canine clinical NDM-5 *E. coli* findings in Europe and this is the first confirmed case of transmission between dogs and humans. While the original source remains unclear, it is likely that either one or both of the dogs had acquired the strain from a human source. Although carbapenems had not been administered to these dogs, frequent exposure to diverse systemic antimicrobials probably facilitated the propagation of the bacteria in the canine host. It is vital for veterinary laboratories to remain alert, and to screen *Enterobacteriaceae* isolates for carbapenem and third-generation cephalosporin resistance. This could be done by using suitable screening disks in testing panels or by using, for example, commercial screening plates for preliminary screening. Isolates with reduced susceptibility to carbapenems should be sent to a reference laboratory for confirmation. Furthermore, the veterinary community should continue to emphasise the importance of prudent antimicrobial use.
